# Dietary Phospholipids and Intestinal Cholesterol Absorption

**DOI:** 10.3390/nu2020116

**Published:** 2010-02-08

**Authors:** Jeffrey S. Cohn, Alvin Kamili, Elaine Wat, Rosanna W. S. Chung, Sally Tandy

**Affiliations:** Nutrition and Metabolism Group, Heart Research Institute, 7 Eliza St. Newtown 2042 NSW, Sydney, Australia; Email: kamili@hri.org.au (A.K.); elaine.wat@gmail.com (E.W.); chungr@hri.org.au (R.W.S.C.); tandys@hri.org.au (S.T.)

**Keywords:** cardiovascular disease, cholesterol, intestine, micelle, phosphatidylcholine, phosphatidylethanolamine, phospholipid, sphingomyelin

## Abstract

Experiments carried out with cultured cells and in experimental animals have consistently shown that phospholipids (PLs) can inhibit intestinal cholesterol absorption. Limited evidence from clinical studies suggests that dietary PL supplementation has a similar effect in man. A number of biological mechanisms have been proposed in order to explain how PL in the gut lumen is able to affect cholesterol uptake by the gut mucosa. Further research is however required to establish whether the ability of PLs to inhibit cholesterol absorption is of therapeutic benefit.

## Abbreviations

HDL, high-density lipoprotein; LDL, low-density lipoprotein; lysoPC, lysophosphatidylcholine; NPC1L1, Niemann-Pick C1-Like1; PC, phosphatidylcholine; PE, phosphatidylethanolamine; PI, phosphatidylinositol; PL, phospholipid; PLA_2_, phospholipase A2; PUFA, polyunsaturated fatty acids; PS, phosphatidylserine; SM, sphingomyelin.

## 1. Introduction

An increased concentration of cholesterol in the blood (*i.e.*, hypercholesterolemia) is widely recognized as a risk factor for coronary artery disease. Reducing plasma levels of total and low-density lipoprotein (LDL) cholesterol by diet, drugs or lifestyle modification is thus of principal importance in treating and preventing cardiovascular disease. In humans, blood cholesterol is derived from two sources. It is either absorbed from the diet by the intestine, or it is synthesized from precursor molecules in the liver. Hepatic cholesterol synthesis can be pharmacologically regulated with statins by inhibiting 3-hydroxy-3-methylglutaryl-CoA reductase, the enzyme responsible for cholesterol synthesis. Statins are very potent lipid-lowering agents and they have been shown to significantly reduce coronary morbidity and mortality [1]. Many patients do not however reach currently defined treatment goals and there is considerable interest in finding additional ways to reduce plasma and LDL cholesterol levels. This has led to the development of a new family of drugs that inhibits intestinal cholesterol absorption. Ezetimibe is a 2-azetidinone compound that reduces cholesterol absorption by inhibiting the protein responsible for cholesterol transport into enterocytes lining the gut lumen, *i.e.*, Niemann-Pick C1-Like1 (NPC1L1) protein. As monotherapy, ezetimibe decreases LDL-C levels by 15–20 percent [2]; in combination with statins it reduces LDL-C by an additional 20–25% [3]. Increasing use of these therapeutic agents has refocussed interest on foods and food components that have the potential to reduce intestinal cholesterol absorption. They include plant sterols, soluble fibres, and saponins [4], which reduce plasma cholesterol and LDL-cholesterol levels by interfering with intestinal cholesterol and bile acid uptake. Different types of phospholipids (PLs) have also been shown to inhibit intestinal cholesterol absorption and the aim of this article is to review our current understanding of the effects of dietary PLs on intestinal cholesterol metabolism.

## 2. Phospholipid Intake in Humans

 The normal dietary intake of PL is 2–8 grams per day, which represents 1–10% of total daily fat intake. Foods with a high PL content include eggs, organ and lean meats, fish, shellfish, cereal grains and oilseeds. Leafy vegetables, fruits and tubers, on the other hand, contain relatively low levels (Table 1) [5]. The most common PL in food is phosphatidylcholine (PC) while other PLs, such as phosphatidylethanolamine (PE), phosphatidylserine (PS) and phosphatidylinositol (PI) are present in much smaller amounts. Sphingomyelin (SM), a phosphorus-containing lipid (more accurately described as a sphingolipid), is present in eggs, meat and fish and is ingested at a level of 0.3–0.4 grams per day. Production of low-fat food products has generally led to a reduction in dietary PL intake. It must not be overlooked however that PL (referred to as lecithin in commercial circles, *i.e.*, a mixture of PC, PE and PI), is widely used as a food additive. Lecithin is a standard ingredient in margarine, and provides consistency of texture to dressings and other creamy products. It acts as an important emulsifier in the manufacture of chocolate and aids in the dispersibility of food powders (e.g., cocoa and whole milk powders). Lecithin is also frequently used as a tin or mould release agent in the bakery and confectionery industry, and can form complexes with starch to improve crumb softness and shelf life of bread. Although levels of lecithin in processed foods are generally low (0.5% or less), their widespread consumption implies that the amount of PL ingested in this form is  not negligible.

**Table 1:** Total lipid and phospholipid content of specific foods.

	Total Lipid (g/100g food)	(mg/100g)						
		Total PL	PC	PE	PI	PS	SM	lysoPC
Egg Yolk	31.8	10,306	6,771	1,917	64	-	486	419
Pig Liver	3.7	2,901	1,688	618	209	38	131	61
Chicken Liver	5.6	2,542	1,120	829	-	146	291	-
Soybeans	20.8	2,308	9,17	536	287	-	-	-
Squid	1.68	1,098	777	114	-	83	102	-
Chicken Breast	1.12	782	391	187	-	100	56	-
Beef	4.1	660	407	207	-	-	46	-
Peanuts	48.5	620	270	50	150	-	-	-
Cod	2.2	580	331	128	23	29	29	6
Spinach	0.3	157	37	36	11	-	-	-
Potato	0.15	76	38	22	12	1	-	-
Carrot	0.3	55	23	15	5	3	-	-
Apple	0.09	40	21	10	6	1	-	-
Cow’s Milk	3.7	34	12	10	2	1	9	-

Abbreviations: PL, phospholipid; PC, phosphatidylcholine; PE, phosphatidylethanolamine; PI, phosphatidylinositol; PS, phosphatidylserine; SM, sphingomyelin; lysoPC, lysophosphatidyl-choline (taken from ref. [5]).

## 3. Phospholipid Digestion and Absorption

The predominant PL in the intestinal lumen is PC, which is the second most abundant lipid in the digestive tract after triglyceride [6]. PC is derived not only from the diet but also from the bile. In fact, the biliary pathway delivers 10–20 g of PL and ~1.5 g of cholesterol to the intestinal lumen per day, which is at least two to four times more than that supplied by the diet. Dietary PC is almost completely absorbed (>90%) by the human intestine, and rapidly appears in plasma lipoproteins and red blood cells [7]. PC digestion occurs in the small intestine, since lingual and gastric lipases are incapable of hydrolyzing PC. Chemical breakdown of PLs is carried out primarily by pancreatic phospholipase A2 (PLA_2_) and other lipases secreted by the pancreas in response to food intake [8]. PLA_2_ is secreted as an anionic zymogen. It is activated by trypsin cleavage and its activity requires the presence of bile salts. It interacts with PLs at the sn-2 position to yield free fatty acid and lysoPC. Both components are taken up by mucosal cells and are resecreted within chylomicrons as newly-formed PL or triglyceride. A proportion of chylomicron PL is subsequently transferred to high-density lipoproteins (HDL), which occurs relatively rapidly, *i.e.*, within 5-6 hours of PL ingestion [9]. Deacylation of lysoPC in the gut lumen is believed to be quite limited and the majority of PL uptake is thus as lysoPC and free fatty acid. Surprisingly, mice deficient in PLA_2_ display no abnormality in dietary PL or triglyceride absorption, suggesting that PLA_2_ may not be essential for PL digestion and that other enzymes can compensate for lack of PLA_2_-mediated PL hydrolysis [10]. 

It is estimated that an adult human on a Western diet ingests 0.3–0.4 grams of sphingolipids per  day [11]. Only a proportion of this is SM, which means that less than 5% of dietary PL is SM. Furthermore, very little dietary SM directly contributes to plasma pools of this PL. The major enzymes responsible for SM degradation in the intestinal lumen and mucosa are alkaline SMase and neutral ceramidase. These enzymes are located in the surface membrane of mucosal cells with catalytic domains facing the outside of the cell. They are responsible for the conversion of SM to ceramide and subsequently to sphingosine and palmitic acid. Ceramide is incompletely absorbed and increasing amounts of SM in the diet leads to an increased proportion of ceramide in the feces [11].

## 4. Cholesterol Absorption

Cholesterol in the gut lumen is derived from the diet (~400 mg/day) and from the bile (~1 g/day). In humans, cholesterol absorption is not complete and on average half of the cholesterol in the gut lumen is absorbed and the remainder is excreted in the faeces. This varies widely between individuals however, and percent absorption varies from 15 to 75% [12]. Individuals also respond differently to changes in dietary cholesterol reflecting the fact that both metabolic and genetic factors regulate cholesterol absorption. The solubilization of cholesterol is very poor in the aqueous environment of the gut and its digestion and absorption is dependent on partitioning into bile micelles. Only nonesterified cholesterol can be incorporated into bile acid micelles and any esterified cholesterol in the diet  (10–15% of total) must be hydrolyzed by cholesterol esterase. Mixed micelles thus contain bile acids, free cholesterol and PL, together with triglyceride, monoacylglyceride, fatty acids and lysophospholipids. These lipid complexes facilitate the transport of cholesterol across the unstirred water layer, which is a diffusional barrier at the intestinal lumen-enterocyte interface. The exact mechanism by which unesterified cholesterol subsequently crosses the brush-border membrane in micelles and is taken up by the mucosal cell remains unclear. For many years, cholesterol absorption was believed to be a simple, energy-independent, passive diffusion process [13]. Cholesterol is absorbed much more efficiently than structurally-related plant sterols however, suggesting that a facilitated mechanism is involved. Three proteins have thus been identified which are believed to be key players in the control of cholesterol absorption, *i.e.*, the cholesterol uptake transporter, Niemann-Pick C1 like 1 (NPC1L1) [14], and the cholesterol efflux transporters: ATP-binding cassette (ABC) proteins ABCG5 and ABCG8 [15]. 

## 5. Phospholipid Effects on Cholesterol Absorption

It is well accepted that the aqueous solubility of cholesterol is extremely low. The transport of cholesterol from the lipid-rich milieu of the intestinal contents to mucosal cells lining the gut lumen is therefore directly dependent on emulsification and micellar solubilization by the detergent-like properties of biliary lipids and the products of dietary lipid lipolysis [16]. Biliary PC is of central importance in this process and cholesterol cannot be effectively solubilized in bile without biliary  PC [17]. Thus intestinal cholesterol absorption relies on adequate amounts of PL in the gut lumen. Contrary to expectation, an excess of PL leads to suppression of cholesterol absorption.

**Figure 1:**
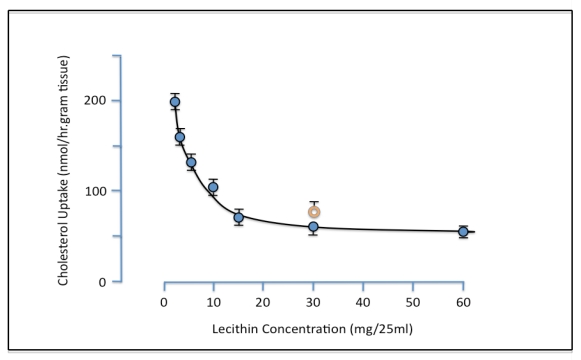
Effect of liver lecithin on intestinal uptake of micellar cholesterol. Everted rat gut sacs were incubated at 37 °C for 1 hr in 25ml micellar solution containing 0.15 mM cholesterol and 4.8 mM bile salt with various amounts of added lecithin. Orange ring symbol represents data obtained with egg lecithin replacing liver lecithin. Each point is the mean of 4–8 experiments. Vertical bars are SE (reproduced from ref. [18]).

Evidence for the ability of PL to inhibit intestinal cholesterol absorption was first provided by Alfred Rampone from the University of Oregon [18]. Bile was known to be essential for cholesterol absorption. The existence of an additional active component in bile was however established when it was shown that uptake of micellar lipids by everted rat intestinal sacs *in vitro* was increased by added bile acid salts but not by whole bile. It was suggested that the inhibitory non-bile salt component might be lecithin and experiments were carried out demonstrating that increasing concentrations of liver lecithin retarded the intestinal uptake of cholesterol [19]. Greater than 50% suppression of cholesterol uptake was observed with the addition of 15 mg (0.8 mM) lecithin, and egg lecithin at a dose of 30mg was found to have a similar effect as a 30 mg dose of liver lecithin (Figure 1). 

Experiments with radioactively labeled lipids and everted rat jejunal gut sacs were subsequently carried out by Rodgers and O’Connor [20]. Dilinoleoyl phosphatidylcholine significantly reduced intestinal uptake of both [^14^C]oleic acid (28% at 2.0 mM) and [^3^H] cholesterol (41% at 2.0 mM). Reduced uptake was not observed with lysoPC, giving rise to the concept that intact PL needed to be present in the gut lumen in order to maintain an effect on cholesterol absorption. It was hypothesized that conversion of PC to lysoPC led to rapid uptake of the lysoPC and disappearance of any inhibitory effect. This was supported by experiments in anaesthetized animals, in which dipalmityl phosphatidylcholine, added to mixed micelles as a diether (*i.e.*, a form resistant to hydrolysis by pancreatic lipase) inhibited intestinal uptake of [^14^C]oleic acid and [^3^H]cholesterol. Further experiments in bile duct-cannulated rats demonstrated that diether PC impaired intestinal absorption of cholesterol to a greater extent than naturally-occurring diester PC in the absence of any significant effect on the absorption of bile salts [21,22].

A number of studies in whole animals have confirmed that native PC exerts an inhibitory effect on intestinal cholesterol absorption. Hollander and Morgan, using a single pass bile-diverted perfusion system in unanaesthetised rats, showed that lecithin (l-α-lecithin) reduced cholesterol absorption in a dose-dependent fashion (*i.e.*, 44, 52 and 54% at doses of 0.5, 1.0 and 1.5 mM respectively) [23]. The absorption, metabolism and excretion of cholesterol was subsequently investigated by O’Mullane and Hawthorne in rats fed diets supplemented with unsaturated lecithin or unsaturated triglyceride [24]. Animals were maintained for 6 weeks on synthetic diets containing 0.5% cholesterol and either 4% by wt corn oil or 6% soya lecithin (providing similar amounts of linoleic acid). Cholesterol absorption measured with a dual isotope ratio method was significantly lower in soya lecithin *vs.* corn oil groups (41.8 ± 4.6% *vs.* 59.7 ± 14.9%, *P* < 0.02). At the same time, fecal output of neutral sterol was significantly higher in soya lecithin-fed animals (22.5 v 15.6 mg/day, *P* < 0.05), providing support for the concept that dietary PC can reduce intestinal cholesterol uptake independent of its linoleic fatty acid content.

The intestinal effects of different forms of PC have been investigated by Jiang *et al. *[25]. Lipid emulsions containing [^14^C]cholesterol were infused via a duodenal catheter into mesenteric lymph duct cannulated rats and the effects of egg PC (EPC), hydrogenated EPC and soy PC (SPC) on the lymphatic appearance of radioactively-labeled cholesterol were compared. SPC had little effect on intestinal cholesterol absorption, whereas egg PC reduced absorption by 20%. The inhibitory effect of hydrogenated EPC was greater than native EPC, suggesting that increased saturation of PL fatty acid acyl groups led to greater inhibition of cholesterol absorption [25]. Intraduodenal infusion of egg sphingomyelin (SM) has also been shown to cause a dose-dependent reduction in the appearance of lymphatic cholesterol. SM lowered the intestinal absorption of cholesterol, alpha-tocopherol and fatty acid but not retinol [26]. Furthermore, milk SM containing fatty acids with a higher degree of saturation and longer chain length was more effective than egg SM in inhibiting cholesterol  absorption [27]. Interestingly, egg SM given orally for periods of three weeks at doses of 0.2 and 0.4% has been found to significantly reduce plasma triglyceride and cholesterol levels in APOE*3Leiden mice fed a Western-type diet. Phytosphingosine, a sphingolipid similar in structure to SM, decreased the absorption of dietary cholesterol and free fatty acids in these animals by 50% and 40% respectively [28].

Although there is good evidence demonstrating an effect of PL on intestinal cholesterol absorption in experimental animals, only a limited number of studies have been carried out in humans. The first of these was conducted by Beil and Grundy [29], who infused human subjects with intraduodenal lecithin or safflower oil with similar fatty acid compositions. The primary objective was to determine whether large amounts of dietary lecithin in the absence of triglyceride could induce the formation of chylomicrons. This was indeed shown to be the case, however a secondary objective was the measurement of cholesterol absorption, which was estimated by measuring the difference in luminal cholesterol concentration at distances 10 cm and 50 cm downstream of the infusion site. The relatively large amount of PL that was infused luminally (150 mg/kg/hr) not only blocked absorption but actually “extracted” cholesterol from the intestinal mucosa, resulting in ‘negative’ absorption values of −45 ± 2% compared to 40 ± 4% obtained in control subjects.

**Figure 2:**
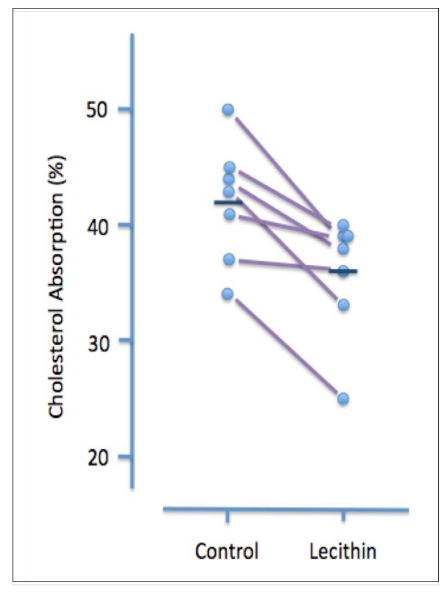
Effect of dietary lecithin (polyenylphosphatidylcholine, 10 grams/day) on cholesterol absorption in hypertriglyceridemic patients. Data are shown for individual patients. During Period I patients were given 7 grams/day of safflower oil and during Period 2 they were given 10 grams/day lecithin (one dose each morning). The amount and composition of fatty acids was equivalent for the two treatments. Cholesterol absorption was measured after two or three weeks by monitoring the ratio of [^14^C] cholesterol to [^3^H] b-sitosterol in stools of patients given oral radioactive sterols. Mean cholesterol absorption (shown by the dark horizontal line) during the control period was 42 ± 2% and during the lecithin treatment period was 36 ± 2%. (reproduced from ref. [30])

A less invasive and more physiological assessment was carried out by the same laboratory several years later [30]. In this study, 10 hypertriglyceridemic patients (nine men and one woman) were treated for five weeks with orally administered safflower oil (7 grams/day), followed by five weeks of treatment with lecithin (10 grams/day). The amount and type of fatty acids in the two oral supplements (given as a single dose each morning) were similar. Cholesterol adsorption was measured during the 2^nd^ or 3^rd^ week of each treatment in seven patients. Although lecithin treatment had no discernible effect on plasma lipid levels, it significantly increased the molar percent of bile acids and decreased the molar percent of lecithin in gall bladder bile. It also had a small but significant inhibitory effect on cholesterol absorption (42 ± 2% v 36 ± 2%, *P* < 0.05) (Figure 2). 

A third study investigated the effect of orally administered lecithin on plasma and whole body lipid metabolism in two normolipidemic subjects and six patients with familial hypercholesterolemia [31]. A fat-modified diet was given for 10 days before 10 grams of polyunsaturated fatty acids (PUFA) were replaced for a further period of 10 days by 18 grams of polyunsaturated PC. The two dietary regimes contained similar calories, and similar amounts of cholesterol and polyunsaturated fatty acids. No significant change was observed in total plasma triglyceride, cholesterol or PL levels when PC was added to the diet. Similarly, no significant change occurred in LDL or HDL cholesterol concentrations. In contrast, fecal sterol analysis in six individuals revealed a consistent increase in fecal neutral sterol excretion (69%) and a negative sterol balance (−1,362 ± 232 *vs.* −793 ± 222 mg/day) during the PC *vs.* PUFA supplementation periods. No evidence was found for a change in the concentration of bile acids, PL or cholesterol in bile. Thus, although cholesterol absorption was not measured directly, indirect evidence was obtained for reduced intestinal cholesterol uptake during PC supplementation.

## 6. Mechanisms for the Inhibitory Effect of PL on Cholesterol Absorption

The aforementioned studies provide consistent evidence that intestinal PLs affect cholesterol uptake from the gut lumen and that dietary PL supplementation can lead to reduced intestinal cholesterol absorption. This raises a fundamental question as to how this biological effect might be mediated. 

There are at least three possible mechanisms (Table 2) that together or alone could explain the effects of luminal PL on intestinal cholesterol absorption. The first possibility is that excess PL interferes with efficient micellar PL hydrolysis, which is a prerequisite for efficient mucosal uptake of cholesterol. Support for this concept is based on the fact that pancreatic secretions are known to be critical for fat absorption, *i.e.*, fat and cholesterol absorption is significantly increased when patients with pancreatic insufficiency are given pancreatic enzymes as a dietary supplement [32]. Bile salt-stimulated carboxyl ester lipase (CEL), also called cholesterol esterase, is one of the major proteins secreted by the pancreas. It has a wide substrate specificity hydrolyzing cholesterol esters, tri-, di- and monoglycerides, and phospholipids [33]. CEL was originally thought to be an important mediator of dietary cholesterol absorption, however, more recent evidence points to a critical role of phospholipase A2 (pPLA_2_). The effect of pPLA_2_ and micellar PL composition on the uptake and metabolism of lipids has been examined in human intestinal Caco-2 cells [34]. PC-containing micelles, though not those containing lysoPC, reduced the uptake, esterification and secretion of cholesterol with little effect on oleic acid absorption. Substitution of half or more of the PC with lysoPC reversed the PC-dependent inhibition. pPLA_2 _also enhanced cholesterol absorption and pPLA_2_-dependent increase in cholesterol absorption was inhibited by a pPLA_2 _inhibitor. Anti-PLA_2_ antibodies have in turn been shown to completely abolish the ability of porcine pancreatic extract to facilitate cholesterol uptake by Caco-2 cells [35], and the PLA_2_ inhibitor (FPL 67047XX) has been shown to retard cholesterol absorption in rats [10]. Together, these data support the concept that absorption of micellar cholesterol by intestinal cells is dependent on elimination of micellar PC and that inhibition of PL hydrolysis leads to reduced cholesterol uptake. One can thus speculate that increased dietary PL may impede or delay normal phospholipase-driven hydrolysis of micellar PL with subsequent downstream effects on  cholesterol absorption.

**Table 2:** Possible mechanisms for inhibition of cholesterol absorption by phospholipid.

1. excess PL interferes with efficient micellar PL hydrolysis - a prerequisite for mucosal uptake of cholesterol
2. surplus PL alters the physicochemical properties of mixed micelles (*i.e.*, their size, composition and/or biological characteristics) resulting in reduced absorption of cholesterol
3. PL acts on the membrane characteristics of enterocytes or has a direct effect on cellular cholesterol transporters that regulate intestinal cholesterol uptake

Cholesterol bioavailability in the intestinal lumen could potentially be reduced by excess PL via a second mechanism. It is feasible that surplus PL could affect the physicochemical properties of mixed micelles (*i.e.*, their size, composition and/or biological characteristics). PL could induce a shift of cholesterol molecules from the micellar phase into lamellar (vesicular) phase from which cholesterol is poorly absorbed - a scenario based on the sequential steps of fat digestion described by  Hernell *et al. *[36]. This model proposes that crude emulsion particles containing predominantly triglyceride enter the duodenum from the stomach. Biliary lipids in various physical states (simple micelles, mixed micelles and vesicles), together with pancreatic lipase and colipase, adsorb to the surface of emulsion particles and lipolysis commences. Lipolytic products are generated and accumulate on the surface of emulsion particles. Increasing amounts of fatty acid and monoglyceride (in the presence of optimal concentrations of bile salts and PL) leads to surface pressures that cause surface lipids to bud off as unilamellar vesicles. These vesicles are converted to micelles in the presence of bile salts and are the ideal physical form for maximal cellular uptake of fatty acids, monoglyceride and cholesterol. It can be speculated that excess PL may impede the conversion of vesicles to micelles. Alternatively, different dietary PLs could reduce micellar solubility of cholesterol thereby impeding the transport of cholesterol to the intestinal mucosa. This possibility is supported by work from Martin Carey’s laboratory in Boston showing that PL acyl chain unsaturation modulates the distribution of lecithin molecular species between mixed micelles and vesicles in model bile [37,38]. Different biochemical techniques revealed that apparent micellar cholesterol solubilities and meta-stable vesicle cholesterol/lecithin molar ratios were 60% and 100% higher, respectively, in bile composed of unsaturated lecithins. At the same time, systems composed of sphingomyelin or the saturated PC dipalmitoyl phosphatidylcholine (DPPC, 16:0–16:0 PC) had poorer cholesterol-solubilizing capacity [39]. Cholesterol uptake by Caco-2 cells was in turn reduced in the presence of milk sphingomyelin, and addition of 0.5% wt/wt milk SM or DPPC to the diet of mice for four days resulted in a significant reduction in cholesterol absorption. The ability of SM to reduce cholesterol absorption has been supported by several studies in rats [26,27,40]. As mentioned earlier, milk SM (containing fatty acids with a higher degree of saturation and longer chain length) was found to be more effective than egg SM in inhibiting cholesterol absorption [27], which supports the concept that the amount and type of SM in the gut lumen can affect the physicochemical characteristics and metabolism of luminal micelles.

A third mechanism pertains to a direct effect of PLs on enterocytes themselves. One can speculate that PLs in the gut lumen may affect the membrane characteristics of enterocytes or have a direct effect on cellular cholesterol transporters thus affecting intestinal cholesterol uptake. Although there is limited data to directly support this mechanism, it is well known that the biological function of cell membranes is very dependent on component PLs and their interaction with intramembrane sterols [41]. Furthermore, the activity of membrane proteins is directly affected by their interaction with membrane PLs [42]. It is thus feasible that dietary PLs could have a direct effect on the absorptive capacity of enterocytes lining the gut lumen. 

## 7. Summary and Conclusions

Both *in vitro* and *in vivo* studies have consistently demonstrated that PLs can inhibit intestinal cholesterol absorption. A number of biological mechanisms have subsequently been proposed to explain how the amount and type of PL in the gut lumen is able to affect cholesterol uptake. Further research is however required to define the extent to which cholesterol absorption can be influenced by dietary PLs in different subjects and different patient groups. The degree to which this biological activity is associated with an effect on plasma lipid levels [43] or hepatic lipid levels [44] needs further investigation. This work will be critical in establishing whether the ability of PLs to inhibit cholesterol absorption is of therapeutic benefit.

## References

[1] Brugts J.J., Yetgin T., Hoeks S.E., Gotto A.M., Shepherd J., Westendorp R.G., de Craen A.J., Knopp R.H., Nakamura H., Ridker P., van Domburg R., Deckers J.W. (2009). The benefits of statins in people without established cardiovascular disease but with cardiovascular risk factors: meta-analysis of randomised controlled trials. BMJ.

[2] Pandor A., Ara R.M., Tumur I., Wilkinson A.J., Paisley S., Duenas A., Durrington P.N., Chilcott J. (2009). Ezetimibe monotherapy for cholesterol lowering in 2,722 people: systematic review and meta-analysis of randomized controlled trials. J. Intern. Med..

[3] Mikhailidis D.P., Sibbring G.C., Ballantyne C.M., Davies G.M., Catapano A.L. (2007). Meta-analysis of the cholesterol-lowering effect of ezetimibe added to ongoing statin therapy. Curr. Med. Res. Opin..

[4] Carr T.P., Jesch E.D. (2006). Food components that reduce cholesterol absorption. Adv. Food Nutr. Res..

[5] Weihrauch J.L., Son Y.S. (1983). The phospholipid content of foods. JAOCS.

[6] Phan C.T., Tso P. (2001). Intestinal lipid absorption and transport. Front Biosci..

[7] Zierenberg O., Grundy S.M. (1982). Intestinal absorption of polyenephosphatidylcholine in man. J. Lipid Res..

[8] van den Bosch H., Postema N.M., de Haas G.H., van Deenen L.L. (1965). On the positional specificity of phospholipase A from pancreas. Biochim. Biophys. Acta.

[9] Tall A.R., Blum C.B., Grundy S.M. (1983). Incorporation of radioactive phospholipid into subclasses of high-density lipoproteins. Am. J. Physiol..

[10] Richmond B.L., Boileau A.C., Zheng S., Huggins K.W., Granholm N.A., Tso P., Hui D.Y. (2001). Compensatory phospholipid digestion is required for cholesterol absorption in pancreatic phospholipase A(2)-deficient mice. Gastroenterology.

[11] Duan R.D., Nilsson A. (2009). Metabolism of sphingolipids in the gut and its relation to inflammation and cancer development. Prog. Lipid Res..

[12] Grundy S.M. (1983). Absorption and metabolism of dietary cholesterol. Annu. Rev. Nutr..

[13] Wilson M.D., Rudel L.L. (1994). Review of cholesterol absorption with emphasis on dietary and biliary cholesterol. J. Lipid Res..

[14] Davis H.R., Altmann S.W. (2009). Niemann-Pick C1 Like 1 (NPC1L1) an intestinal sterol transporter. Biochim. Biophys. Acta.

[15] Sabeva N.S., Liu J., Graf G.A. (2009). The ABCG5 ABCG8 sterol transporter and phytosterols: implications for cardiometabolic disease. Curr. Opin. Endocrinol. Diabetes Obes..

[16] Iqbal J., Hussain M.M. (2009). Intestinal lipid absorption. Am. J. Physiol. Endocrinol. Metab..

[17] Carey M.C., Small D.M. (1978). The physical chemistry of cholesterol solubility in bile. Relationship to gallstone formation and dissolution in man. J. Clin. Invest..

[18] Rampone A.J. (1972). The effects of bile salt and raw bile on the intestinal absorption of micellar fatty acid in the rat in vitro. J. Physiol..

[19] Rampone A.J. (1973). The effect of lecithin on intestinal cholesterol uptake by rat intestine in vitro. J. Physiol..

[20] Rodgers J.B., O'Connor P.J. (1975). Effect of phosphatidylcholine on fatty acid and cholesterol absorption from mixed micellar solutions. Biochim. Biophys. Acta.

[21] O'Connor P.J., Rodgers J.B. (1976). The effect of diether phosphatidylcholine on the enterohepatic circulation of biliary sterols. Biochim. Biophys. Acta..

[22] O'Connor P.J., Loiudice T.A., Bochenek W., Rodgers J.B. (1978). Effect of diester and diether phosphatidylcholine on intestinal absorption of neutral and acidic sterols. Am. J. Dig. Dis..

[23] Hollander D., Morgan D. (1980). Effect of plant sterols, fatty acids and lecithin on cholesterol absorption in vivo in the rat. Lipids.

[24] O'Mullane J.E., Hawthorne J.N. (1982). A comparison of the effects of feeding linoleic acid-rich lecithin or corn oil on cholesterol absorption and metabolism in the rat. Atherosclerosis.

[25] Jiang Y., Noh S.K., Koo S.I. (2001). Egg phosphatidylcholine decreases the lymphatic absorption of cholesterol in rats. J. Nutr..

[26] Noh S.K., Koo S.I. (2003). Egg sphingomyelin lowers the lymphatic absorption of cholesterol and alpha-tocopherol in rats. J. Nutr..

[27] Noh S.K., Koo S.I. (2004). Milk sphingomyelin is more effective than egg sphingomyelin in inhibiting intestinal absorption of cholesterol and fat in rats. J. Nutr..

[28] Duivenvoorden I., Voshol P.J., Rensen P.C., van Duyvenvoorde W., Romijn J.A., Emeis J.J., Havekes L.M., Nieuwenhuizen W.F. (2006). Dietary sphingolipids lower plasma cholesterol and triacylglycerol and prevent liver steatosis in APOE*3Leiden mice. Am. J. Clin. Nutr..

[29] Beil F.U., Grundy S.M. (1980). Studies on plasma lipoproteins during absorption of exogenous lecithin in man. J. Lipid Res..

[30] Kesaniemi Y.A., Grundy S. M. (1986). Effects of dietary polyenylphosphatidylcholine on metabolism of cholesterol and triglycerides in hypertriglyceridemic patients. Am. J. Clin. Nutr..

[31] Greten H., Raetzer H., Stiehl A., Schettler G. (1980). The effect of polyunsaturated phosphatidyl-choline on plasma lipids and fecal sterol excretion. Atherosclerosis.

[32] Vuoristo M., Vaananen H., Miettinen T.A. (1992). Cholesterol malabsorption in pancreatic insufficiency: effects of enzyme substitution. Gastroenterology.

[33] Hui D.Y., Howles P.N. (2002). Carboxyl ester lipase: structure-function relationship and physiological role in lipoprotein metabolism and atherosclerosis. J. Lipid Res..

[34] Homan R., Hamelehle K.L. (1998). Phospholipase A2 relieves phosphatidylcholine inhibition of micellar cholesterol absorption and transport by human intestinal cell line Caco-2. J. Lipid Res..

[35] Mackay K., Starr J.R., Lawn R.M., Ellsworth J.L. (1997). Phosphatidylcholine hydrolysis is required for pancreatic cholesterol esterase- and phospholipase A2-facilitated cholesterol uptake into intestinal Caco-2 cells. J. Biol. Chem..

[36] Hernell O., Staggers J.E., Carey M.C. (1990). Physical-chemical behavior of dietary and biliary lipids during intestinal digestion and absorption. 2. Phase analysis and aggregation states of luminal lipids during duodenal fat digestion in healthy adult human beings. Biochemistry.

[37] Cohen D.E., Carey M.C. (1991). Acyl chain unsaturation modulates distribution of lecithin molecular species between mixed micelles and vesicles in model bile. Implications for particle structure and metastable cholesterol solubilities. J. Lipid Res..

[38] van Erpecum K.J., Carey M.C. (1997). Influence of bile salts on molecular interactions between sphingomyelin and cholesterol: relevance to bile formation and stability. Biochim. Biophys. Acta..

[39] Eckhardt E.R., Wang D.Q., Donovan J.M., Carey M.C. (2002). Dietary sphingomyelin suppresses intestinal cholesterol absorption by decreasing thermodynamic activity of cholesterol monomers. Gastroenterology.

[40] Nyberg L., Duan R.D., Nilsson A. (2000). A mutual inhibitory effect on absorption of sphingomyelin and cholesterol. J. Nutr. Biochem..

[41] Quinn P.J., Wolf C. (2009). The liquid-ordered phase in membranes. Biochim. Biophys. Acta.

[42] Dowhan W., Bogdanov M. (2009). Lipid-dependent membrane protein topogenesis. Annu. Rev. Biochem..

[43] Knuiman J.T., Beynen A.C., Katan M.B. (1989). Lecithin intake and serum cholesterol. Am. J. Clin. Nutr..

[44] Cohn J.S., Wat E., Kamili A., Tandy S. (2008). Dietary phospholipids, hepatic lipid metabolism and cardiovascular disease. Curr. Opin. Lipidol..

